# Ecological Vulnerability Assessment Based on Fuzzy Analytical Method and Analytic Hierarchy Process in Yellow River Delta

**DOI:** 10.3390/ijerph15050855

**Published:** 2018-04-25

**Authors:** Chunsheng Wu, Gaohuan Liu, Chong Huang, Qingsheng Liu, Xudong Guan

**Affiliations:** State Key Laboratory of Resources and Environmental Information System, Institute of Geographic Science and Natural Resources Research, Chinese Academy of Sciences, Beijing 100101, China; wuchsh0118@163.com (C.W.); huangch@lreis.ac.cn (C.H.); liuqs@lreis.ac.cn (Q.L.); guanxd@lreis.ac.cn (X.G.)

**Keywords:** ecological vulnerability assessment, fuzzy analytical method, analytic hierarchy process, fuzzy membership, yellow river delta

## Abstract

The Yellow River Delta (YRD), located in Yellow River estuary, is characterized by rich ecological system types, and provides habitats or migration stations for wild birds, all of which makes the delta an ecological barrier or ecotone for inland areas. Nevertheless, the abundant natural resources of YRD have brought huge challenges to the area, and frequent human activities and natural disasters have damaged the ecological systems seriously, and certain ecological functions have been threatened. Therefore, it is necessary to determine the status of the ecological environment based on scientific methods, which can provide scientifically robust data for the managers or stakeholders to adopt timely ecological protection measures. The aim of this study was to obtain the spatial distribution of the ecological vulnerability (EV) in YRD based on 21 indicators selected from underwater status, soil condition, land use, landform, vegetation cover, meteorological conditions, ocean influence, and social economy. In addition, the fuzzy analytic hierarchy process (FAHP) method was used to obtain the weights of the selected indicators, and a fuzzy logic model was constructed to obtain the result. The result showed that the spatial distribution of the EV grades was regular, while the fuzzy membership of EV decreased gradually from the coastline to inland area, especially around the river crossing, where it had the lowest EV. Along the coastline, the dikes had an obviously protective effect for the inner area, while the EV was higher in the area where no dikes were built. This result also showed that the soil condition and groundwater status were highly related to the EV spatially, with the correlation coefficients −0.55 and −0.74 respectively, and human activities had exerted considerable pressure on the ecological environment.

## 1. Introduction

Ecological vulnerability (EV), which was first brought into ecological theory as “Ecotone” by Clements in the 1900s [1], was not taken seriously until “Silent Spring” was published in 1960s. However, there is still no EV definition that is accepted by all scholars, who reference different theories according to their research contents. By combining previous studies, we conclude that EV can be described through such characteristics as weak ecological stability, weak anti-interference ability, and low recovery capability when the ecosystem or subsystem is suffering external disturbances [2]. The assessment of EV can be designed to generate the degree an ecosystem is sensitive to losing its functionality when exposed to environmental or anthropogenic pressures. A series of environmental problems, such as global warming, glacial melt, flood, drought, and pollution, make the ecological environment extremely sensitive and cause the vulnerable areas to spread rapidly, seriously affecting ecological sustainable development [3], which make EV assessment necessary.

EV studies’ development is in accompaniment with the improvements of different ecological theories and scientific methods, which has become quantitative compared to early studies [4,5]. The research scales are also refined from countries or big river basins to one lake or one mine [6,7]. The research fields include forests, grasslands [8], coastal zones [9], agro-grazing ecotones, and others, but for river deltas—which combine the characteristics of rivers, lands, and oceans—EV studies are scarcer [10]. River deltas contain many ecosystem types, especially numerous wetlands, which offer habitats to wild animals and increase biodiversity. Therefore, it is highly valuable to carry out EV studies when deltas are threatened by human activities and natural hazards.

Various methods have been employed in EV assessments, including fuzzy analysis model [11], principal component analysis (PCA) [12], gray relation method [13], and analytic hierarchy process (AHP) [14]. However, any method has its drawbacks: the AHP is affected heavily by artificial subjective factors, and the gray relation method is complicated in processing, while useful information loss has yet to be solved in PCA. Another important aspect in EV assessment is the selection of indicators [15], for which, scholars have set some models or frameworks, including “pressure-state-response (PSR)” [16], “exposure-sensitivity-adaptability (ESA)” [17], and “driving force-pressure-state-impact-response-management (DPSIRM)” [18]. Moreover, indicators are increasingly comprehensive, as various socio-ecological problems emerge.

The Yellow River Delta (YRD), which is formed from the sediment carried by the Yellow River, has abundant natural landscapes, and provides important biodiversity protection and ecological buffer functions for inland. Nevertheless, abundant land and oil resources have brought human activities to the region, which have exceeded the YRD’s capacity. Therefore, assessment of EV for the sustainable development of YRD is required. Several scholars have completed relative studies in this area, such as Wolters [19], who used questionnaires to assess the educational status, the income, and the occupation types of rural residents and their responses to floods, droughts, and other natural disasters, and later analyzed the vulnerability of the rural environment qualitatively. Other studies tended to use the AHP to evaluate the EV of YRD, but the artificial subjective factor remained unsolved [20,21].

This study intended to combine the fuzzy analysis model and AHP to evaluate the EV of YRD in a method called fuzzy analytic hierarchy process (FAHP). The triangular fuzzy number of fuzzy set theory was brought into the pairwise comparison matrix of AHP, as it could better accommodate the imprecision and ambiguity that occurred in criterial judgement process, and it also could reduce the influence of the artificial subjective factors in comparison to the traditional AHP. On the contrary, the clear indicator framework of AHP simplified the fuzzy analysis process. Additionally, the EV fuzzy memberships of all indicators were generated as the basis of classification, which could avoid the strict boundary in the numerical classification process.

The primary object of this study was to evaluate the EV of the YRD. Other objectives were (1) to verify the feasibility of FAHP in the EV assessment of YRD, and (2) to analyze the spatial heterogeneity according to the final spatial distribution of EV, and subsequently, provide scientific and effective suggestions for the sustainable development of YRD.

## 2. Materials and Methods

### 2.1. Study Area

**Figure 1:**
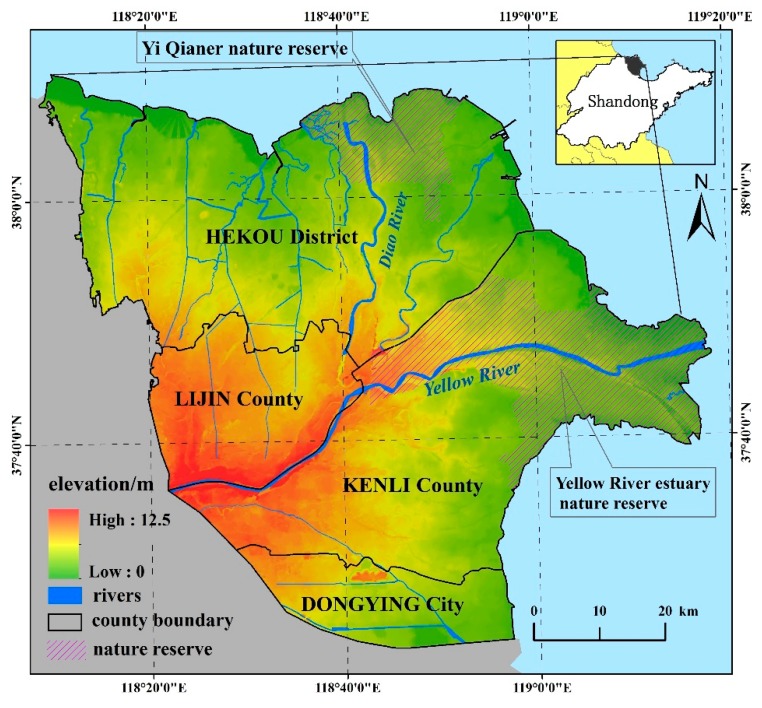
Location and elevation of the study area.

The Yellow River Delta (YRD) is located in the northeast of Shandong province, China with coordinates between 37°22′ N–38°04′ N and 118°14′ E–119°05′ E, and a total area of approximately 5062.59 km^2^, surrounded by the Bo Sea in the north and east (Figure 1). The terrain is gentle, with the highest elevation being approximately 12.50 m in the southwest, and the lowest, at approximately 0 m in the northeast. As a result of previous river migrations, various microtopography features are interspersed in the area, including flat grounds, high lands, tidal flats, and depressions. The YRD has a temperate continental monsoon climate, and the rainy season is from June to September. However, the annual mean evaporation is greater than the annual mean precipitation, which is approximately 1885.00 mm and 537.40 mm, respectively. The main soil type is gleyic solonchaks with high salinization and a high sand proportion, resulting in low soil quality. The natural vegetation is largely consisting of herbaceous plants, such as bulrush, tamarix, cogon, and suaeda. Two state reserves are located in the north (Yi Qian Er nature reserve) and east (Yellow River estuary nature reserve) of the study area. Human activity is frequent, which included farmland development, industry construction, and oil resource exploration, and all of which has led to landscape fragmentation.

### 2.2. Indicator System Establishment 

Reasonable and scientifically evaluated indicators should be selected for the reliability of the assessment process and its result, and they must address the main environmental problems of the area, which can be summarized as follows:

- Ocean tides and storm surge intrusions have destroyed the coastal land environment directly.
- The concentrated rainfall results in frequent flooding, and the higher annual mean evaporation limits the vegetation growth.
- The shallow groundwater and the high groundwater mineralization make the soil naturally saline. In addition, the farming and farmland abandonment because of production drawdown have resulted in more serious secondary soil salinity.
- Except for the two natural reserves, human activities have influenced the natural landscape heavily, leading to natural wetlands loss, vegetation coverage decrease, and net primary productivity decline.
- The use of fertilizers and pesticides and industrial pollutant discharge have added the EV of the study area.

Based on the above problems, this study selected 21 assessment indicators from groundwater status, soil condition, land use, landform, vegetation coverage, meteorological conditions, ocean influence, and social economy. All indicators were listed as Table 1:

### 2.3. Data Collection and Processing

Groundwater status: The annual average groundwater level and groundwater mineralization of 16 wells previously set in the study area were collected. The two indicators were proven to be related to the soil salinity and land degradation [22], which was conducive to EV. The spatial distribution over the study area of the two indicators was generated by ordinary kriging model.

Ocean influences: The direct performances of ocean tides and storm surge intrusions were coastline change. Therefore, 13 coastlines from 1984 to 2014 were generated from Landsat TM images based on the mean high water model [23]. The two adjacent coastlines were overlapped to obtain the spatial changes over the coast, and the change frequency at every location was regarded as the coastal erosion index. The distance to coastline of inland locations could represent the probability of being eroded by seawater, as most coastlines were soft sediments. It was calculated according to the coastline in 2014 using Euclidean distance model.

Meteorological conditions: 13 meteorological monitoring sites in and around the study area were selected for the meteorological data collection, and the annual average daily temperature and annual average daily rainfall of the 13 sites were retrieved from the China Meteorological Data Service Center (http://data.cma.cn/), as they were important for the vegetation growth. Next, the annual average ≥10 °C accumulated temperature (*AT*) and the annual average rainfall were generated, and used to calculate the aridity index (*K*) using the following equation [24]:

K=(0.16×AT)/AR.

*AR* represents the annual average rainfall at temperature ≥10 °C. The spatial distributions of the meteorological indicators were generated using ordinary kriging model based on the 13 sites.

Socio-economic data: This type of data was accessed in numerical and tabular form from the 2015 yearbook of China, 2015 yearbook of Shandong Province and 2015 yearbook of Dongying city. The population density and the Gross Domestic Product (GDP) density were calculated based on the statistical data of population and gross domestic product and administrative area. The spatial distributions of the population density and the GDP density over the whole study area were generated using a model created by Liao Shunbao [25] that refers to the “population-residence-influence” framework.

Vegetation: The normalized difference vegetation index (NDVI), derived from the Landsat 8 OLI image acquired on 30 September 2014, was used to generate the vegetation status based on the followed equation:

NDVI=(NIR−R)/(NIR+R)

*NIR* and *R* represented the near-infrared band and red band of the image, respectively.

The soil salinity, soil quality, and land-use status in 2014 were based on the study results of Wu Chunsheng [26,27,28]. The elevation, microtopography features (including depressions, flat grounds, tidal flats, and high lands), soil types, and soil textures were provided by the Data Center for Resources and Environmental Sciences, Chinese Academy of Sciences (RESDC) (http://www.resdc.cn/).

Other data sets, including the land reclamation rate, the human interference index, the road and canal densities, were all generated from the land-use data set, and the unit of road and canal densities was km/km^2^.

All data sets were projected to WGS84-based Transverse Mercator orthographic projection coordinate system, and were resampled to a 30 m × 30 m spatial resolution, and the final maps had the same resolution as the data sets.

### 2.4. Methodology

#### 2.4.1. Fuzzy Logic Model

Fuzzy membership values are regarded as the probability of one indicator belonging to some grades instead of some fixed numbers [29]. The common fuzzy membership function is bell-shaped:

$$MF_{x_{i}}=\left[1/\left(1+{\left(\left(x_{i}-b\right)/d\right)}^{2}\right)\right]$$

where 0 < $MF_{x_{i}}$ ≤ 1; $MF_{x_{i}}$ is the fuzzy membership value of indicator i; $x_{i}$ is the value of i; and d is the transition width of i, which is set as the indicator value difference when the membership values are 0.5 and 1. Index b is the indicator value when the membership value is 1 [30,31].

Setting suitable ranges of indicators according to the study object is necessary in the model. The membership value can be gained through functions when the indicator value is among the range; otherwise, the membership value is 0 or 1. The suitable ranges of indicators are determined through consulting previous study results, documentations or standard specifications, and this process is important and difficult. In addition, the quantitative and qualitative indicators should be discriminatory because of the lack of numbers in qualitative indicators.

Through consulting the literature, previous study results [32,33,34] that relate to Yellow River Delta and professional books [35], the suitable ranges, and index *b* and *d* of quantitative indicators could be set as Table 2, meanwhile, the membership values of qualitative indicators would be listed directly (Table 3 and Table 4).

#### 2.4.2. Fuzzy Analytic Hierarchy Process

The FAHP has been used in previous EV studies. For example, Liu analyzed the spatial-temporal change of the EV in the Sanjiangyuan Region from 1990 to 2010 based on the FAHP [36]. The uses of FAHP in different studies were distinguishing, especially in pairwise comparison matrix and weight calculation processes [37]. This study attempted to use the fuzzy trigonometric function to assign the pairwise comparison values to decrease the error during matrix establishment.

First, the AHP pairwise comparison matrix was established, and the values 1 to 9 were assigned as Table 5 [38]. The values were modified in accordance with the advice of experts from hydrology, climatology, soil science, and ecology, and the matrix passed the consistency test [39,40]. Then, the values were replaced with the abscissa values of the fuzzy trigonometric function suggested by Kahraman [41].

Second, the weights were generated through the following processes. The importance value of indicator *i* compared to indicator *j* could be assumed as (*l_ij_*, *m_ij_*, *u_ij_*) [42], where *l*, *m*, *u*, were the abscissa values of the fuzzy trigonometric function. Conversely, the importance value of indicator *j* compared to indicator *i* could be set as (1/*u_ij_*, 1/*m_ij_*, 1/*l_ij_*) (Table 5). The fuzzy cumulative extension value of *i* ($M_{g_{i}}$), which represented the importance of indicator *i* compared to all indicators, was calculated using the equation

$$M_{g_{i}}=\left(l_{i1}+l_{i2}+\cdots +l_{in},\;m_{i1}+m_{i2}+\cdots +m_{in},\;u_{i1}+u_{i2}+\cdots +u_{in}\right)\;=\left(\overset{n}{\underset{\alpha =1}{\sum }}l_{i\alpha },\overset{n}{\underset{\alpha =1}{\sum }}m_{i\alpha },\overset{n}{\underset{\alpha =1}{\sum }}u_{i\alpha }\right)$$

Then, the fuzzy cumulative extension value of pairwise comparison matrix could be gained:

$$\overset{n}{\underset{i=1}{\sum }}\overset{n}{\underset{\alpha =1}{\sum }}M_{g}=\left(\overset{n}{\underset{i=1}{\sum }}\overset{n}{\underset{\alpha =1}{\sum }}l_{i\alpha },\;\overset{n}{\underset{i=1}{\sum }}\overset{n}{\underset{\alpha =1}{\sum }}m_{i\alpha },\;\overset{n}{\underset{i=1}{\sum }}\overset{n}{\underset{\alpha =1}{\sum }}u_{i\alpha }\right)$$

Also, the fuzzy synthetic extension value of *i* ($S_{i}$), which represented the synthetic importance proportion of indicator *i* in the matrix, could be calculated from the equation

$$S_{i}=M_{g_{i}}\times {\left[\overset{n}{\underset{i=1}{\sum }}\overset{n}{\underset{\alpha =1}{\sum }}M_{g}\right]}^{-1}=\left(l_{i},\;m_{i},\;u_{i}\right)$$

where *n* was the indicator number. For $S_{i}$ and $S_{j}$, their comparison value could be expressed as

$$V\left(S_{i}\geq S_{j}\right)=hgt\left(S_{i}\cap S_{j}\right)=\mu_{S_{i}}\left(a\right)=\{\begin{array}{c} 1,\;\;\;\;\;\;\;m_{i}\geq m_{j}\\ \;0,\;\;\;\;\;\;\;\;l_{j}\geq u_{i}\;\\ \frac{l_{j}-u_{i}}{\left(m_{i}-u_{i}\right)-\left(m_{j}-l_{j}\right)},\;others \end{array}$$

The $d_{i}{}^{\prime }$ and $w^{\prime }$ were the transitional value and matrix:

$$d_{i}{}^{\prime }=min\left(V\left(S_{i}\geq S_{k}\right)\right),\;i\neq k,\;k=1,\;2,\cdots n\;w^{\prime }={\left(d_{1}{}^{\prime },d_{2}{}^{\prime },\cdots ,d_{n}{}^{\prime }\right)}^{T}$$

The weight matrix *w* was generated by standardizing $w^{\prime }$:

$$w={\left(d_{1},d_{2},\cdots ,d_{n}\right)}^{T}$$

In these calculation processes, comparison matrix standardization was necessary, while the phenomenon $V\left(S_{i}\geq S_{k}\right)=0$ might appear frequently, which would make the result unreasonable.

The pairwise comparison matrix of two level indicators was created following the processes of FAHP (Table 5). Moreover, the final weights calculated following the equations above were listed in Table 6, in which the relative weight replaces the weight of second-level indicator in the relative first-level indicator, while the synthesis weight replaces the weight of second-level indicator in all second-level indicators.

The indicator weight expressed its contribution to the EV. The soil condition was extremely important to the ecological security and stability according to its high weight 0.31. Inversely, the gentle landform over the study area had a relatively small effect on the spatial distribution of the EV relatively, as shown in Table 6.

#### 2.4.3. EV Assessment

The weighted sum model was used in the final evaluation:

$$EVI=\overset{n}{\underset{i=1}{\sum }}A_{i}\times w_{i}$$

where *EVI* is the vulnerability value, $w_{i}$ is the weight of indicator i, and $A_{i}$ is the membership value of indicator i. In addition, the natural break point method was utilized to rank the comprehensive grades of the EV, as it is based on the principle of minimizing the sum of the variances at each level to select the grading breakpoints, and the breakpoint itself is a good boundary for classification.

## 3. Results

**Figure 2:**
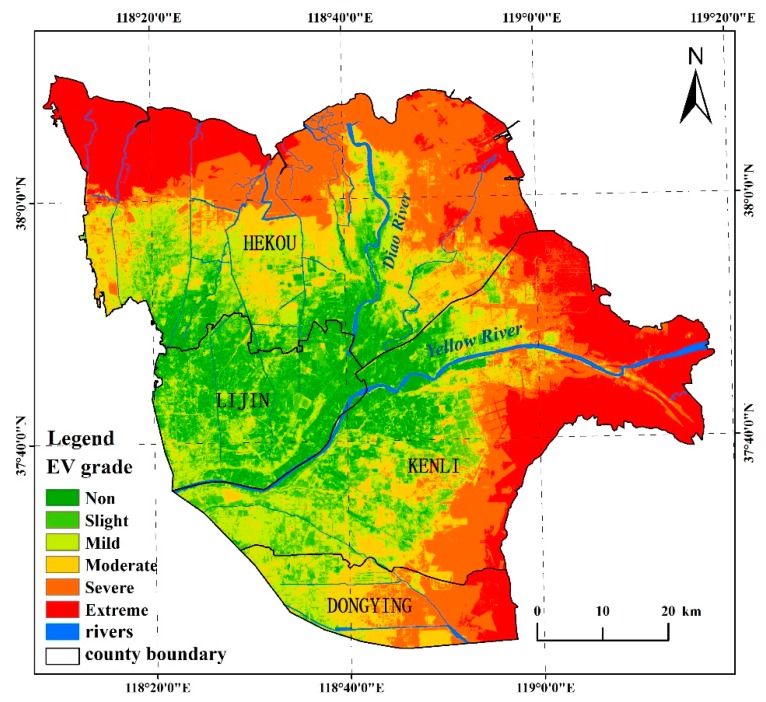
Spatial distribution of the ecological vulnerability in the study area.

The synthetically fuzzy membership values of EV and its grades in YRD generated through the weighted sum model were shown in Figure 2, and the statistic of each grade was listed in Table 7.

Figure 2 and Table 7 showed that the closer to the coastline, the higher the EV was, especially around the Yellow River estuary and northwestern coast of the study area. The EV was lowest around the crossing of Yellow and Diao rivers. The comprehensive spatial distribution of the EV was reasonable, and the statistic was credible compared with other similar studies.

Severe grade was the most widely type with area 1113.25 km^2^, which was 22.03% of the total study area, and mainly distributed in the north coast. Next was the extreme grade with area 952.85 km^2^, and it was nearer to coastline than the others were, except for the coastal area of Yi Qianer nature reserve. The moderate, mild, slight, and non-vulnerable grades distributed from the periphery to inland successively, and the non-vulnerable grade had the smallest area 464.17 km^2^.

The overlay of dominant land-use types and EV was generated as Table 6 showed that the land-use status had a high contributory degree to EV with a high weight, which was slightly lower than the soil condition. The statistics were listed in Table 8:

Table 8 showed that the farmland had the largest area, which mainly distributed in slight grade, while a small portion of the farmland also distributed in extreme and severe grades. Compared to the farmland, the saline land distributed equally in each grade except for the non-vulnerable, and the severe grade had the largest area with 290.10 km^2^. Almost the entire coastal beach belonged to the extreme grade, and the grassland and forest distributed equally in all the grades.

**Figure 3:**
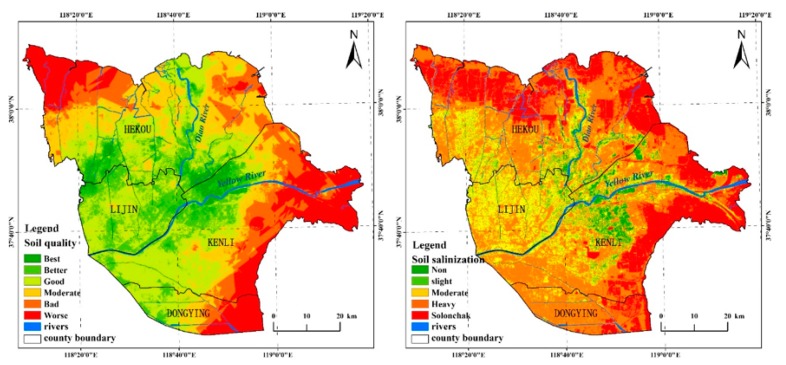
Spatial distributions of the soil quality and the soil salinization in study area.

From the indicator weights shown in Table 6, we could conclude that the soil condition affected the evaluation results deeply, among which, the soil salinity and the soil quality were most influential, because their weights were higher. In contrast to the research results of Wu Chunsheng [28] regarding the soil quality assessment of the YRD (Figure 3), the spatial distributions of soil salinity and soil quality were similar with the EV, and the Pearson’s correlation coefficient between the soil quality and EV was −0.55. The YRD usually had a high EV value where the soil quality was poor, such as at the mouth of the Yellow River and the northwest and southeast of the study area. Conversely, the soil quality was rich where the Yellow and Diao rivers meet, but this area was not vulnerable.

Other indicators that were germane to EV included the land-use status and groundwater status. The human interference index was weighted largely in the land-use status, resulting from heavy farmland development and artificial wetland construction.

**Figure 4:**
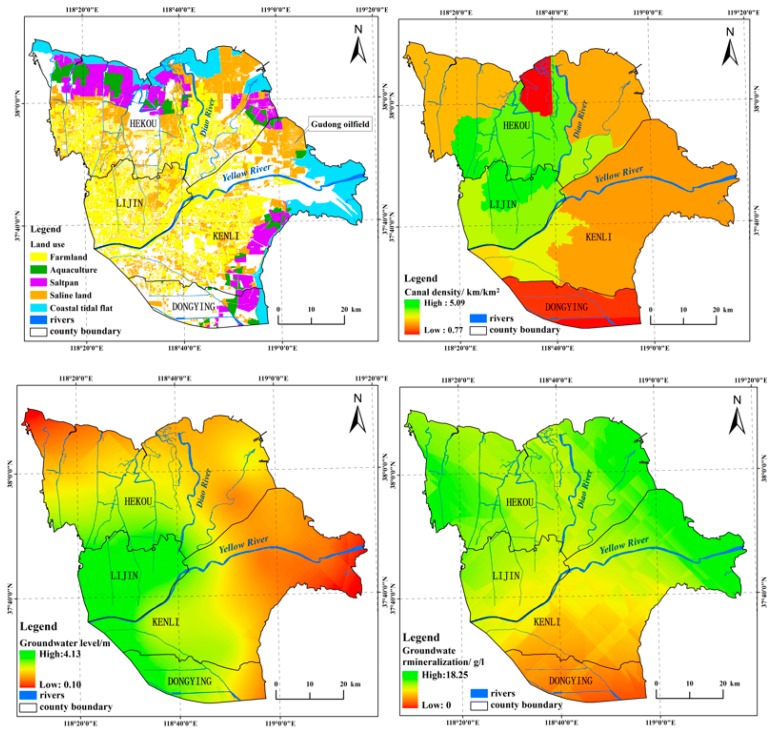
Spatial distributions of main land-use status and groundwater status in study area.

Figure 4 showed that the groundwater level had similar spatial distribution characteristics to EV, and the Pearson’s correlation coefficient was −0.74. Furthermore, the high degree of mineralization in groundwater was in favor of topsoil salinization. In particular, when the groundwater level was shallower, the salinization was heavier. The low vegetation cover resulted in low net primary productivity, causing the ecological environment difficult to recover when it was destroyed by external disturbances. 

Additionally, the EV along the coastline was obviously different as shown in Figure 2, and based on the field survey, we could affirm that the dikes were very helpful for ecological environment protection. From the north of the Gudong oilfield to the west of the Diao River estuary, different types of dikes were constructed, which prevented intrusions of ocean tides and storm surges effectively, ensured vegetation growth and enhanced the ecological environment’s stability.

## 4. Discussion 

According to the results above, we found that the spatial distribution of EV was coincided with previous assumption and actual natural conditions, which indicated that the FAHP and fuzzy logic model were applicable for EV assessment in YRD. Compared to other similar study results [20,43,44,45], the methods decreased anthropogenic influences as the fuzzy trigonometric function processes were more objective, while the weights in other studies were all manually specified. The use of the membership function was not only helpful for the quantitative evaluation, but also made the grading more flexible, which finally resulted in a more detailed analysis and successive spatial distribution.

The soil condition, groundwater, and land-use status were main influencing factors of EV, according to the study results. Therefore, how to improve these factors was the main goal to reduce the vulnerability of ecological environment. However, the spatial distribution map of the main land use (Figure 4.) and the statistics provided by the overlay between the main land-use types and EV (Table 8) showed that a fraction of farmland was developed in high EV areas, which was not good for the maintenance and protection of ecological environment. This type of farmland was usually abandoned as its soil quality was low, which would result in secondary salinization. Additionally, the construction of aquacultures and saltpans destroyed the original evolutionary direction of the natural environment, increasing the degree of the region’s vulnerability. Based on this situation, it is necessary to reduce or eliminate artificial disturbances on land with high ecological vulnerability, or to de-salt existing cultivated land, such as designing some salt drains and gutters. Constructing dikes was beneficial to improve groundwater status in the study area, which in turn could weaken the vulnerability of the ecological environment in most areas, but it was not suitable for the Yellow River estuary because of the habitat protection, which needed the interaction between ocean and inland, and decreasing the anthropogenic influence should be the best treatment. Therefore, considering different treatment methods according to different coastline types was beneficial to ecological environment protection.

Previous studies using the FAHP were different in selection of pairwise comparison method and weight calculation model, but these methods have not been compared, and the fitness of different models should be explored in follow-up studies. Additionally, this study was only based on the data in the year 2014, making the analysis monotonous and limiting the argument. For a deeper analysis of the ecological environment characteristics, spatial dynamic changes in EV should be determined based on multiphase data; however, the lack of preliminary data before 2014 made it impossible to do so. Therefore, future research will depend on the continuity of later data, and the applicability of selected indicators must be given sufficient attention.

## 5. Conclusions

This study completed the EV assessment of YRD based on the FAHP and fuzzy logic models, and results demonstrated that the combination of the two models was useful for the assessment. The spatial distribution of all vulnerability grades was regular. Vulnerability decreased from the coastline to inland, and the ecological environment was especially vulnerable around the estuary of Yellow River and the northwest and southeast of YRD. However, the ecological environment condition was excellent where the Yellow and Diao rivers meet. Generally, the spatial distribution pattern of EV was reasonable. As a special indicator in YRD, the soil salinity was heavily weighted; obviously, it affected the regional ecosystem stability severely. The soil quality, as represented by soil fertilizer, was affected the ecological environment more heavily, and the Pearson’s correlation coefficient was −0.55 between the soil quality and EV. The groundwater level affected soil salinity directly, and further affected the ecological environment indirectly with a Pearson’s correlation coefficient −0.74. Additionally, human activities, such as farming, the construction of aquacultures and saltpans, and oil exploration, brought adverse effects to the ecological environment in YRD; however, the construction of dikes along the coastline was beneficial for the ecological environment stability. For further analysis of the spatial distribution of EV and advice regarding its ecological environment protection, the dynamic change of EV was needed in further studies.

## Figures and Tables

**Table 1:** Two levels of indicators selected for ecological vulnerability (EV).

First-Level Indicator	Second-Level Indicator	First-Level Indicator	Second-Level Indicator
Groundwater status	Groundwater level	Land use	Land reclamation rate
	Groundwater mineralization		Human interference index
Soil condition	Soil type		Land cover
	Soil texture		Canals density
	Soil quality	Vegetation	NDVI
	Soil salinity	Socio-economic	Population density
Ocean influences	Distance to coastline		Roads density
	Coastal erosion index		GDP density
Meteorological Conditions	Annual average rainfall	Landform	Elevation
	≥10 °C accumulated temperature		Microtopography features
	aridity index		

**Table 2:** Parameter values in the fuzzy membership function.

Indicators	Suitable Range	Trends	*b*	*d*
Ground water level/m	1–3	Negative	1	1
Groundwater mineralization/g/l	2–30	Positive	30	20
Soil quality	0.3–0.7	Negative	0.3	0.2
Soil salinity/%	0.1–0.6	Positive	0.6	0.2
Distance to coastline/km	2–30	Negative	2	23
Coastal erosion index	0–0.83	Positive	0.83	0.33
Land reclamation rate	0.03–0.5	Positive	0.5	0.2
Human interference index	0.5–1	Positive	1	0.2
Canals density/km/km^2^	1–5	Negative	1	2
NDVI	0.1–0.6	Negative	0.1	0.35
Population density/population/km^2^	100–1500	Positive	1500	1000
Roads density/km/km^2^	0.2–1.5	Positive	1.5	0.9
GDP density/10 k yuan/km^2^	50–2500	Positive	2500	2000
Annual average rainfall/mm	400–1000	Negative	400	300
≥10 °C accumulated temperature/°C	4300–4600	Negative	4300	200
Aridity index	1.0–1.7	Positive	1.7	0.35
Elevation	4.0–8.0	Negative	8.0	2

**Table 3:** Membership values of different land-use types.

Land-Use Type	Membership	Land-Use Type	Membership
River	0	Canal	0.7
Forest	0.1	Inland tidal flat	0.7
Farmland	0.2	Dike	0.7
Garden plot	0.2	Mine zone	0.8
Pond	0.3	Port	0.8
Reservoir	0.3	Saline land	0.9
Grassland	0.4	Saltpan	0.9
Traffic land	0.4	Aquaculture	0.9
Residence	0.4	Coastal tidal flat	1
Marshland	0.6		

**Table 4:** Membership values of different soil characteristic and microtopography.

Soil Texture	Membership	Soil Type	Membership	Microtopography	Membership
Water	0	Water	0	Water	0
Medium loam	0.2	Moisture soil	0.3	High lands	0.2
Light loam	0.5	Damp soil	0.6	Flat	0.4
Weight loam	0.5	Coastal saline soil	0.9	Flood land	0.6
Clay	0.6			Depressions	0.8
Sandy loam	0.7			Tidal flats	1

**Table 5:** Importance values setting of pairwise comparison for fuzzy analytic hierarchy process (FAHP).

Linguistic Scales of Importance	AHP Number Scale	Triangular Fuzzy Scale	Reciprocal Triangular Fuzzy Numbers
Just equal	1	(1,1,1)	(1,1,1)
Equally important	1	(1/2,1,3/2)	(2/3,1,2)
Weakly more important	3	(1,3/2,2)	(1/2,2/3,1)
Strongly more important	5	(3/2,2,5/2)	(2/5,1/2,2/3)
Very strongly more important	7	(2,5/2,3)	(1/3,2/5,1/2)
Absolutely more important	9	(5/2,3,7/2)	(2/7,1/3,2/5)

**Table 6:** Weights of all indicators for EV.

First-Level Indicator	Weight	Second-Level Indicator	Relative Weight	Synthesis Weight
Groundwater status	0.19	Groundwater level	0.50	0.095
		Groundwater mineralization	0.50	0.095
Soil condition	0.31	Soil type	0.09	0.0279
		Soil texture	0.07	0.0217
		Soil quality	0.40	0.124
		Soil salinity	0.44	0.1364
Ocean influences	0.05	Distance to coastline	0.22	0.011
		Coastal erosion index	0.78	0.039
Meteorological conditions	0.04	Annual average rainfall	0.57	0.0228
		≥10 °C accumulated temperature	0.30	0.012
		Aridity index	0.13	0.0052
Land-use status	0.23	Land reclamation rate	0.06	0.0138
		Human interference index	0.50	0.115
		Land cover	0.17	0.0391
		Canals density	0.27	0.0621
Vegetation	0.12	NDVI	1.00	0.12
Socio-economic	0.04	Population density	0.50	0.02
		Roads density	0.19	0.0076
		GDP density	0.31	0.0124
Landform	0.02	Elevation	0.68	0.0136
		Microtopography features	0.32	0.0064

**Table 7:** Numerical statistics of the different EV grades.

EV Grade	Membership Range	Area/km^2^	Proportion/%
Non	0.16–0.32	464.17	9.19
Slight	0.32–0.41	820.40	16.23
Mild	0.41–0.50	901.42	17.84
Moderate	0.50–0.59	801.42	15.86
Severe	0.59–0.67	1113.25	22.03
Extreme	0.67–0.78	952.82	18.85

**Table 8:** Area of main land-use types distributed in each EV grade (unit: km^2^).

	Grades	Non	Slight	Mild	Moderate	Severe	Extreme	Total
Types								
Grassland		10.28	23.07	45.03	48.47	37.28	3.46	167.59
Inland tidal flat		3.92	6.61	5.32	2.38	5.72	11.08	35.03
Farmland		327.23	405.06	226.98	104.66	46.42	4.06	1114.40
Coastal tidal flat		-	-	0.00	0.74	117.91	359.50	478.15
Saline land		26.97	116.86	189.54	188.38	290.10	102.41	914.26
Garden plot		8.26	10.00	2.71	0.34	0.01	-	21.33
Forest		21.05	34.25	32.90	30.07	48.29	24.24	190.80
Total		397.71	595.85	502.49	375.04	545.73	504.75	2921.57
